# Effects of reflection-through-action on collaborative clinical reasoning skills in an emergency dental simulation

**DOI:** 10.1186/s41077-026-00463-6

**Published:** 2026-07-27

**Authors:** Lauren Glowski, Nikolas Grotewold, Sarah Tomaka, Theodora E. Danciu, Vitaliy Popov

**Affiliations:** 1 Department of Learning Health Sciences, School of Information Victor Vaughan, University of Michigan Medical School, 217, 1111 Catherine St, Ann Arbor, MI 48109 USA; 2 Department of Neurology, University of Michigan Medical School, 1500 E Medical Center Dr, Ann Arbor, MI 48109 USA; 3 University of Michigan School of Dentistry, 1011 N University Ave, Ann Arbor, MI 48109 USA; 4 University of Michigan School of Information, 105 S State St, Ann Arbor, MI 48104 USA

**Keywords:** Simulation-based learning, Medical education, Dental education, Clinical simulation, Reflective pause, Debriefing, Clinical reasoning, Learning transfer, Reflection-through-action

## Abstract

**Background:**

Simulation-based training is a central component of healthcare education. However, most current instructional designs emphasize post-simulation debriefing, while reflection during the simulation itself, termed reflection-through-action, remains understudied. Prior empirical evidence suggests that structured reflective pauses may enhance learners’ metacognitive regulation and diagnostic and intervention clinical reasoning, though whether these learning gains transfer to subsequent, novel clinical scenarios is unknown. This study examined whether reflection-through-action during an initial emergency simulation enhances the translation of technical skills and collaborative clinical reasoning practices in a subsequent emergency simulation among dental students.

**Methods:**

In this mixed-methods study, 110 dental students were randomized into teams and completed two sequential medical emergency simulations. Six experimental teams received structured reflection-through-action during the first simulation, consisting of a guided pause with written prompts and a structured PARSE (Pause–Analyze–Reflect–Strategize–Execute) debrief, while six control teams completed the simulation without reflective pauses. Outcomes accounted for prior knowledge and team familiarity and included expert-rated technical and non-technical performance, self-reported learning confidence, and behavioral process-level analyses using Epistemic Network Analysis (ENA) of team conversations during the second simulation.

**Results:**

Controlling for prior knowledge and team familiarity, no differences were seen in expert-rated technical or non-technical performance or in self-reported learning confidence between groups. However, ENA revealed important structural differences in the collaborative clinical reasoning displayed during the second simulation. Learners who engaged in reflective pauses demonstrated stronger connections between suggesting/requesting action, planning interventions, and intervening actions, indicating a greater connection between diagnostic reasoning and treatment management. In contrast, control teams showed weaker connections between planning and action, with conversations emphasizing procedural explanations, disconnected suggestions, and unfocused patient history questions.

**Conclusion:**

A structured reflective pause did not produce immediate differences on traditional performance metrics but revealed meaningful changes in collaborative clinical reasoning including how teams organized diagnostic, planning, and action during a subsequent emergency simulation. These findings suggest that reflective pauses reshape the structure of a team’s clinical reasoning and support near (contextually similar) and proximal (temporal) learning transfer. ENA offers a more sensitive and analytic approach than traditional performance-based outcomes for detecting differences in team-based collaborative clinical reasoning.

**Supplementary Information:**

The online version contains supplementary material available at https://doi.org/10.1186/s41077-026-00463-6.

## Background

In high-stakes healthcare contexts, clinicians must continuously adapt their actions based on incomplete information and evolving patient conditions – all under time pressure and frequently in coordination with others [1, 2]. Effective performance in these settings depends not only on individual clinical knowledge but on how teams collectively gather and interpret patient information, assign roles, and translate shared understanding into coordinated action [3]. Simulation-based training has become an essential component of evidence-based learning within health professional education, offering learners the opportunity to develop these essential clinical skills by practicing decision-making without the potential for patient harm [4, 5]. Within simulation, structured reflection is an established mechanism for bridging conceptual knowledge with clinical action, enabling learners to apply learned information to dynamic and uncertain clinical contexts [6–8]. However, despite widespread adoption of clinical simulation and mostly positioning pre- and post-simulation reflection at the boundaries of tasks, instructional designs rarely incorporate structured within-simulation reflection systematically, with even fewer studies examining whethere its learning effects persist beyond the initial task [9–12].

The reflective practice literature distinguishes between related but conceptually different forms of reflection. Schön [13, 14] described reflection-on-action as retrospective consideration of practice after an event has occurred, often supported by a more knowledgeable other or facilitator, through which practitioners analyze experience, improve their understanding, and refine future action [13–15]. In contrast, reflection-in-action refers to the capacity to examine one’s reasoning and adjust subsequent action(s) while performing. Reflection-in-action can be viewed as a developmental capacity to recognize patterns, reason, problem solve and adjust decisions in real time without interrupting the flow of action. Both forms, as originally conceived, are individual cognitive processes. Yet, in a team setting, reflection involves collective sense-making in which team members externalize individual interpretations, engage in reflective discussion, and formulate improvement strategies [16, 17].

This collective dimension of reflection can be viewed through the concept of team reflexivity. Defined as the extent to which team members overtly reflect upon, co-construct meaning, and communicate about their team’s objectives, strategies, and processes [18–20]. It is distinct from individual reflection in that it requires communication, is externally observable, and functions as a shared information-processing activity that precedes collective adaptation. At the clinical level, brief structured intraoperative briefings (i.e., surgeon-initiated pauses to communicate operational status, next steps, and potential problems to the full operating room team) reduced postoperative mortality and unplanned reoperations across four surgical departments [21, 22]. These findings indicate that structuring team reflection during action phases has measurable effects for both learning and patient outcomes [23].

In simulation, however, novice learners may lack self-regulatory and metacognitive skills necessary for independently determining *when to pause* and *how to reflect* effectively and require external scaffolding to build this capacity [15, 24, 25]. Sullivan et al. described clinical simulation as an ideal learning environment to foster this capacity through reflection-through-action [26]. This is a form of guided team reflection that briefly interrupts a simulation at a critical juncture, allowing learners to use the mechanisms of reflection-on-action, externalize their reasoning, reassess decisions, anticipate risks, and plan subsequent action before the scenario resumes. With facilitation, learners are offered opportunities to self-correct while learning is still occurring, and before errors become reinforced through continued action [27]. Current evidence suggests that deliberately inserting pauses during simulation to encourage reflection may enhance learning in terms of identifying clinical deterioration, recognizing a need for and seeking assistance, modifying actions, and strengthening cognitive reasoning [11, 28]. This approach builds on prior work supporting deliberate reflection, which found that guided reflection on decision-making enhances diagnostic learning and problem solving in dynamic clinical contexts [29]. Despite this theoretical base, investigation into the function of reflection-through-action during high-fidelity simulation remains limited [30].

What further remains poorly understood is whether reflection-through-action produces effects that extend beyond the immediate simulation and whether those effects operate at the team as well as the individual level. Collaborative clinical reasoning, as used throughout the paper, refers to the process by which clinicians collectively gather, interpret, and integrate patient information (e.g., patient history, etiology of symptoms) and guide their treatment decisions (e.g., medication administration) through coordinated communication and teamwork. Near transfer refers to the application of skills to a contextually similar but novel task, while immediate transfer refers to the application of recently acquired capacities close in time to their acquisition [31]. Deliberate individual reflection has been shown to promote more analytical approaches to future clinical problems and to strengthen diagnostic performance for adjacent but novel conditions [10, 29]. Whether an equivalent effect operates at the team level, specifically, whether reflection-through-action affects the collaborative clinical reasoning teams bring to a subsequent, unfamiliar scenario, remains an open question. Conventional simulation outcome measures, including expert-rated performance scores and self-reported measures, tend to capture time to critical actions or task completion rather than the reasoning processes of teams, limiting what prior work has been able to detect [24, 30].

This study addresses current gaps by examining how a structured reflective pause during an initial emergency scenario in high-fidelity clinical simulation impacts collaborative clinical reasoning during a subsequent, unfamiliar scenario. Using a randomized mixed-methods design, we compare teams receiving structured reflection-through-action with teams completing standard simulation practice. Our primary research questions were as follows: (1) do structured reflective pauses improve team performance in a subsequent, novel scenario, and (2) which collaborative clinical reasoning practices, if any, differ between the conditions?

## Methods

### Participants

Following approval from the institutional research ethics board, 110 dental students (65 females, 45 males, mean age = 23, SD = 3.05) voluntarily participated in the study. All students were enrolled in the University of Michigan School of Dentistry. Students self-identified as predominantly White and Asian and represented diverse geographic backgrounds, including 21 U.S. states as well as international origins from Canada, China, and South Korea. Common undergraduate majors included Biology, Microbiology, and Biochemistry. Five of the participants had earned a master’s degree prior to entering dental school.

### Learning scenarios

**Fig. 1:**
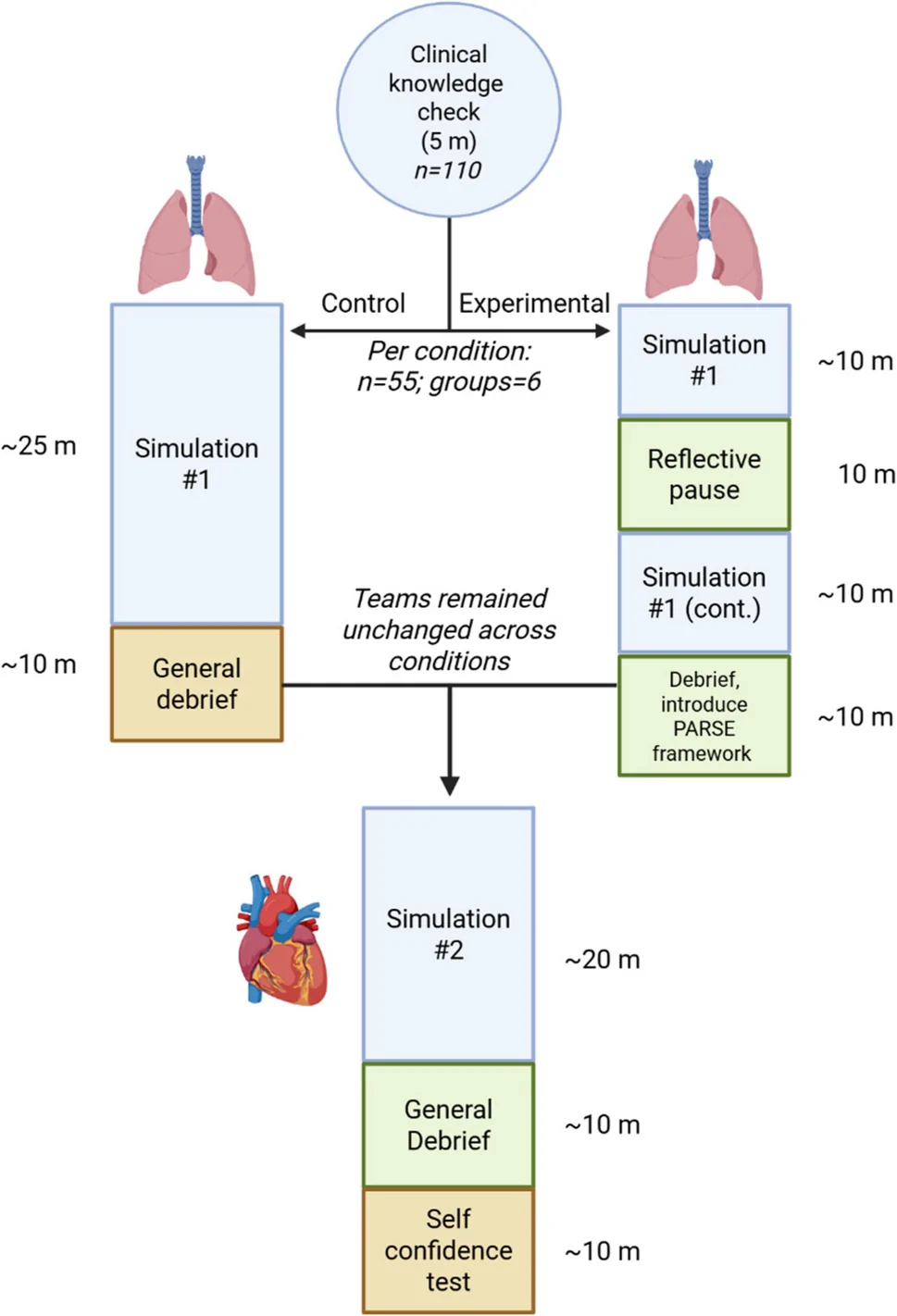
Overview of the study design. Students were randomized to control or intervention groups and completed two sequential simulation scenarios. The intervention group received a reflective pause during the first respiratory scenario and PARSE-guided debrief between scenarios. Performance in the second simulation was used to assess transfer of learning

Participants completed two sequential medical emergency simulations: a respiratory scenario followed by a cardiovascular scenario (see Fig. 1). To examine transfer of learning, all analytic comparisons focused on team performance during the second (cardiovascular) simulation.

As dental students begin their clinical experiences during their second year, they must be prepared to recognize and respond to respiratory and cardiovascular emergencies that may arise in patient care settings. Thus, the simulation content was designed to reinforce concurrent coursework while supporting the development of essential clinical skills, including application of biomedical knowledge, recognition of medical emergencies, clinical decision-making, and patient safety. In this way, the activity served as a curricular bridge between didactic instruction and early clinical practice.

#### Respiratory simulation scenario

This respiratory simulation scenario centers around a 53-year-old male with severe chronic obstructive pulmonary disease (COPD) and an extensive smoking history, who arrives for planned mandibular tooth extractions in preparation for a removable prosthesis. The scenario emphasizes the importance of thorough pre-procedural assessment, including updating medical history, verifying medications, and evaluating the patient’s current respiratory status, which is noted to be worse than usual on the day of the procedure. Key features such as pursed-lip breathing, tripod positioning, and wheezing, as well as abnormal vital signs and supplemental oxygen use, prompt students to recognize a likely COPD exacerbation possibly triggered by infection, medication nonadherence, or environmental factors.

During the procedure, administration of a bronchodilator (albuterol) stabilizes the patient, allowing for a cautious, single-tooth extraction, with specific consideration to avoid a fully reclined position and bilateral anesthesia. However, aspiration of a tooth leads to acute respiratory distress and hypoxemia, with exam findings consistent with airway obstruction and unilateral wheezing. Students are guided to identify an aspiration event based on clinical and anatomical cues and to prioritize an emergency medical referral. The case reinforces key concepts in managing dental patients with significant pulmonary disease, patient safety, and medical emergency response, as integral to dental practice.

#### Cardiovascular simulation scenario

The scenario centers on a 72-year-old male with a significant cardiac and metabolic history, presenting for extraction of an unrestorable tooth followed by implant planning. His medical history includes insulin-dependent diabetes with renal and retinal complications, stable angina managed with nitroglycerin, well-controlled hypertension, hypercholesterolemia, and a longstanding smoking habit. Despite cessation of daily aspirin prior to the procedure, students are prompted to assess the relevance of each condition and its implications for dental care, focusing on the importance of clarifying the stability of angina and updating medical records. During the simulation, “Mr. Angus” develops acute chest pain while undergoing a routine extraction under local anesthesia with epinephrine. His symptoms, including sweating, hypertension, tachycardia, and decreased oxygen saturation, require immediate evaluation for a potential myocardial infarction. The scenario challenges students to differentiate cardiac etiologies, initiate appropriate use of sublingual nitroglycerin, and recognize the need for emergency referrals when symptoms persist, integrating principles of safe dental practice and medical emergency response for patients with complex systemic diseases.

### Study design

Students were randomly assigned to twelve groups, consisting of approximately nine students per group. Group size was determined by curricular logistics, including the large number of students in the second-year dental class and the need to accommodate all participants across two simulation days with available faculty and simulation resources. All groups completed both the respiratory and cardiovascular simulation scenarios, with six groups randomly assigned to the intervention condition and six to the control condition. The intervention consisted of a structured reflection-through-action activity embedded within the first (respiratory) simulation, while control groups completed the simulation without the pause. After the respiratory scenario debrief with faculty, all teams completed the second (cardiovascular) simulation, during which team performance was evaluated to assess collaborative clinical reasoning and transfer of learning (Fig. 1).

After the first scenario, control groups participated in the standard faculty-led debrief, which reviewed case events, clinical management, and expected emergency-response actions using the standardized instructor guide. The guide included case background, learning objectives, scripted prompts, and criteria for successful completion. Unlike the intervention groups, control groups did not use the PARSE framework to structure reflection before the second simulation. PARSE was discussed with all students as part of the educational debrief after study activities were completed, ensuring instructional equity while avoiding influence on the control group before the assessed transfer scenario. To support intervention implementation fidelity, instructors were provided with a standardized document outlining the case background, target learning objectives, and scripted prompts for the simulation. Criteria for successful simulation completion were also predefined in the instructor guide, including administering nitroglycerin and contacting emergency services. In addition to this written material, the instructional team held several meetings prior to implementation to review the cases, align expectations, and clarify how the simulation should unfold to promote consistent delivery among instructors.

### Reflection-through-action intervention

This study consisted of two consecutive medical emergency scenarios: first, a respiratory scenario, followed by a cardiovascular scenario. Each scenario required active problem-solving, as diagnostic and treatment information was not explicitly provided. The intervention included two components: (1) a structured reflective pause (10 min.) embedded within the respiratory simulation, and (2) a facilitated post-simulation debrief using the PARSE framework conducted prior to the cardiovascular scenario.

The reflective pause occurred at a critical learning juncture [11] during the respiratory simulation, following initial stabilization with albuterol administration and before the subsequent aspiration event. This timing positioned the pause between the pre-procedural assessment and stabilization and the intra-procedural emergency management. During this 10-minute pause, students individually recorded their clinical reasoning, anticipated complications, and planned interventions using Post-it notes. Students then collaborated in pairs to synthesize their responses before resuming the simulation. This reflective pause aimed to help students externalize and evaluate their risk mitigation strategies before the aspiration emergency, promoting transfer from anticipatory clinical reasoning to crisis management.

#### Reflective prompts

Students in the experimental group were instructed to use color-coded Post-it notes to answer four questions during a 10-minute reflection period, which occurred during the first (respiratory) simulation, prior to intervention decision-making (use of inhaler). The questions were as follows: *1. How well have we worked together as a team thus far? What could help us be more effective (e.g. information sharing*,* communication*,* role clarity*,* speaking up/challenging*,* asking for/offering help*,* awareness of goals/priorities*,* etc.)? 2. What would make this patient have a medical emergency? 3. What are the signs and symptoms that can alert you that the patient is at risk for a medical emergency? 4. How can you prevent/address this medical emergency?”* After individual reflection, students then collaborated in pairs to synthesize their responses before resuming the simulation.

#### PARSE framework

The PARSE framework (Supplementary Material 1: Appendix A) guided students’ reflections during the post-simulation debrief. This framework encompasses five domains: Patient history/vitals, Anticipate risks, Recognize symptoms, Strategize prevention, and Execute response. Instructors provided students with the PARSE table and discussed how the framework is relevant to the simulation and real-life clinical scenarios. Students then used the framework to strategize for the second case by focusing on the teamwork-focused question: *What should we do differently or focus on next time in terms of teamwork (e.g. communication*,* seeking/offering help*,* role clarity*,* information sharing)? Be clear about who will do that and how this will help our team be successful in the next simulation case.*

### Instruments

#### Pre- and post-simulation knowledge survey

Participants individually completed pre- and post-simulation assessments to measure respiratory and cardiovascular knowledge (Supplementary Material 1: Appendix B).

#### Familiarity measure

Prior to participating in the simulation, participants completed the Inclusion of Other in the Self (IOS) Scale [32], which asked participants to provide familiarity ratings on a seven-point scale for each group member. The seven options are accompanied by pictorials measuring their relationship with another participant, as visualized by increasingly overlapping circular figures. A rating of 1, or completely disjointed circles, indicates no familiarity and a rating of 7, or nearly fully overlapping circles, indicates a very close relationship.

#### Performance measure

Performances by each of the 12 dental student teams were evaluated independently by two dental faculty members. Faculty evaluators rated student performance across two domains: non-technical skills (Leadership/Roles, Teamwork, Communication, and Situational Awareness/Conflict Resolution) [33] and technical skills (Medical History, Evaluating Information, and Patient Management) [17]. Two course instructors developed criteria to assess technical skills based on established institutional competencies for managing this cardiovascular crisis in dentistry. Each subcategory was graded on a 3-point scale, where 1 indicated below-expected performance, 2 represented satisfactory performance, and 3 denoted above-expected performance (Table 1).

**Table 1:** Performance rubric used to evaluate student teams during a simulated dental emergency. Each domain was scored on a three-point scale (Beginner = 1, Intermediate = 2, Advanced = 3) based on predefined criteria reflecting observable team behaviors

Domain	Beginner (1)	Intermediate (2)	Advanced (3)
Non-technical skills			
Medical History	Critical safety checks are incomplete or missing	The team obtains most relevant medical history and confirms some safety elements, but not all key criteria.	Comprehensive history including explicit confirmation of nitroglycerin availability, absence of chest pain at rest, and patient stability immediately prior to the procedure. Procedures clearly explained.
Evaluating Information	Clinical reasoning is limited with information gathered but not synthesized into a working hypothesis. Recognition of symptoms occurs only after patient deterioration.	The team collects relevant clinical information and performs partial interpretation. Reasoning develops reactively during events.	Integrates medical history, symptoms, and risk factors to form a proactive working hypothesis. Anticipates potential complications and clearly articulates rationale for decisions.
Patient Management	Team displays delayed or confused response to patient deterioration; key interventions not prioritized or improperly executed.	Appropriate management steps taken after symptoms develop, but response delayed or only partially organized. Preventive counseling limited.	Prompt and organized response including cessation of procedure, timely medication administration, activation of emergency response, monitoring, and patient support. Provides clear preventive counseling to the patient (e.g., smoking cessation).
Technical Skills			
Leadership/Roles	No clear leader or task delegation present; team generally operates through unstructured discussion.	Informal leadership or partial task assignment is present but roles are not clearly defined.	Clear leadership with explicit task delegation, coordination, and summaries of team priorities and next steps.
Teamwork	Tasks completed inconsistently with limited coordination; roles unclear.	Team members collaborate and adapt to changing conditions but coordination is informal.	Highly coordinated teamwork with explicit task ownership, prioritization, and closed-loop communication.
Communication	Team communication unclear or inconsistent; instructions or reasoning poorly conveyed during critical moments.	Generally clear and supportive communication with patient; team communication occasionally disorganized under stress.	Clear, calm, and patient-centered communication. Team dialogue is structured and supportive with explicit confirmation of actions.
Situational Awareness/Conflict Resolution	The team reacts to problems only after they escalate; limited monitoring of evolving patient status.	The team recognizes and responds to changes in patient condition but primarily after patient symptoms appear.	Anticipates potential complications, continuously monitors patient status, and responds rapidly while maintaining patient reassurance and team coordination.

#### Confidence self-reports

After the simulation, dental students were asked to complete a questionnaire reflecting on their self-confidence after the simulation, simulation design, and educational practices [34–36]. The instrument consisted of eight items rated on a 5-point Likert scale (1 = strongly disagree to 5 = strongly agree). Items addressed students’ confidence in mastering simulation content, perceived relevance to dental clinical practice, quality of instructional resources, and understanding of their own learning responsibilities. Question 8 was reverse coded prior to analysis to account for differences in wording.

### Analyses

Baseline statistical differences in team prior knowledge and team familiarity were assessed using Mann-Whitney U tests. Expert rated team-level technical and non-technical performances and individual self-reported confidence scores were compared between conditions using independent two-tailed t-tests. All comparisons were performed at the team level except for self-reported confidence scores, which were compared descriptively at the individual level across conditions. Because individual participants were nested within teams, the individual-level self-reported confidence analysis should account for within-team non-independence [37]. Statistical analyses were performed and graphed in GraphPad Prism (version 10.0.0; GraphPad Software, San Diego, CA, USA).

#### Epistemic network analysis

For analysis of collaborative clinical reasoning behaviors, we employed Epistemic Network Analysis (ENA; R package version 1.7.0; Shaffer 2018) [38]. ENA is a method that allows representing temporal co-occurrence by sliding a window over the sequence of coded events, even with smaller sample sizes, and generating weighted connections between codes within that window. ENA enables comparison of association structures both visually and via summary statistics that reflect the structure of connections among coded utterances [39]. This makes it possible to identify significant differences among groups of units and explain those differences in terms of the associations between them.

We defined the units of analysis as all lines of data associated with each student team subsetted by condition (experimental or control). We used a “moving stanza” model. Following the selection method proposed by Ruis and colleagues [40], we set the window size to 3. The resulting networks are aggregated for all conversations for each unit of analysis. In this model, we aggregated networks using a binary summation in which the networks for a given conversation reflect the presence or absence of the co-occurrence of each pair of codes (see Table 2).

**Table 2:** Coding scheme for analysis of collaborative clinical reasoning behaviors. Primary codes, sub-codes, definitions, and representative examples adapted from Schmutz et al. [12]

Primary Code	Sub-Code	Definition	Examples
Seeking Information from the Patient	Eliciting Information	The care team asked relevant questions regarding this patient’s angina, including: do you experience chest pain at rest? Is your chest pain relieved by nitroglycerin?	“Do you have any sort of cough or any trouble breathing, any sort of respiratory issues that we should be aware of?” “[UH] it says here you had a physical last month, is that right?”
	Explaining Procedures	The care team made today’s procedures clear to the patient: anything being done or that will be done to the patient.	“We’re just going to go over some basic medical and dental history before we begin, just to make sure that we can provide you with appropriate care today. Is that okay?” “If you don’t mind, we’re gonna take [UH] a little glucose measurement on your finger if that’s okay.”
Seeking Information from the Team	Sharing and Requesting Information	A care team member provided or requested information from other team members about the current event.	“Did anyone ask him about his aspirin?” “Do you guys have any other [UH] information that you guys want to gather?”
	Suggesting and Requesting Action	The care team verbalized initial thoughts regarding how to proceed with patient care unrelated to the cardiovascular event.	“So patient’s blood pressure is slightly high, but do you guys think it’s okay to *proceed* with the extraction, for this number?” “Let’s recheck the blood pressure.”
Evaluating Information	Developing a Working Hypothesis	The care team discussed and formulated a working hypothesis about the patient’s cardiovascular disease, risk factors and change in status.	“And he has angina, so that must be something related to like heart attacks.” “Do you think he might have aspirated his tooth in there?”
	Recapping Information and Providing Rationale	The care team brought various pieces of information together and provided a summary, or the care team offered a reason to support a decision or intervention.	“And also he has high blood pressure and high heart rate, that’s the main concern right now, cause the SpO2 is normal.” “[UM] we were just talking about aspirin, and like, that’s kind of typically, like the aspirin is to thin the blood a little bit so it flows better.”
Planning and Acting on a Decision	Planning an Intervention	The care team suggested action-based interventions to manage the patient’s care related to the cardiovascular event.	“Should he take his angina, his medication?” “And we could like, monitor his vitals.”
	Smoking Cessation Counseling	The care team encouraged smoking cessation/nicotine replacement.	“…no matter the work that we do today, it’s really important to kind of maintain the health of your teeth, and stopping smoking is gonna be necessary to do that.” “Have you tried any [UH] supplementary approaches, like tobacco chewing gum?”
	Taking Action to Implement Intervention	The care team took action to implement a decision or intervention made by the team, occurring before or after the cardiovascular event.	“I’m calling EMS” “I’m giving him two puffs of nitroglycerin, and we’ll see how it goes”

Networks were visualized using network graphs, where nodes corresponded to the codes, and lines connecting the nodes reflected the relative frequency of co-occurrence, or connection, between two codes. Node size indicates the frequency of occurrence of the code, and the thickness of edges shows the strength of the relationship. We statistically tested differences between the conditions in the cardiovascular scenario by applying Wilcoxon rank sum tests to ENA scores that allowed us to evaluate whether students in the experimental conditions displayed distinct connection patterns, and link statistical differences to specific network structures.

#### Coding scheme

A coding scheme (Table 2) was developed using elements of the Team Reflection Behavioral Observation (TuRBO) framework created by Schmutz et al. [12]. Using the coding scheme as a guide, individual codes were then modified for specificity and more accurate capture of the simulation scenario and learning objectives. For example, “Actively Inviting Input” from the TuRBO coding scheme was modified to distinguish patient interactions from team interactions, therefore codes “Patient: Eliciting Information” and “Team: Requesting/Sharing Information” were developed. Additionally, a distinction was made between “Plan: Intervention” and “Action: Intervention,” as a means of specifying the team’s generation of ideas toward treating the cardiovascular event, and the team’s decision to act on a chosen intervention. All coded transcripts were iteratively reviewed by two research team members with clinical background to ensure consistent coding.

#### ELAN

Following the transcription of all video recordings, two first authors (LG and NG) collaboratively annotated all twelve videos together using consensus-based approach [41] and the coding scheme (Table 2). Phrase-level utterances were transcribed and coded using EUDICO Linguistic Annotator (ELAN) [42]. This consensus-based approach for a relatively small number of recordings enabled us continuous calibration throughout the coding process, reducing the risk of coding drift over time. The annotators reviewed each video together, referenced the transcripts, and explicitly discussed their reasoning for each coding decision until agreement was reached. The annotators also documented their coding decisions systematically in an evolving codebook to ensure consistency throughout the analysis.

## Results

### Prior knowledge surveys

In the 5-question pre-simulation knowledge quiz, the control group (*N* = 55) and experimental group (*N* = 55) demonstrated similar pre-clinical knowledge levels (control: 1.7 ± 0.88; experimental 1.7 ± 1.08; *p* = 0.906). For the two questions specific to cardiovascular knowledge, the control group underperformed in comparison to the experimental group (control: 0.15 ± 0.41; experimental: 0.42 ± 0.63; *p* = 0.0087). Although the experimental group demonstrated slightly higher baseline cardiovascular knowledge on the pre-simulation quiz, overall pre-clinical knowledge levels were comparable across groups.

### Team familiarity measures

Participant familiarity ratings, derived from the IOS Scale [20], showed modest variability across the twelve teams. Average team familiarity scores ranged from 2.06 to 4.11 on the seven-point scale. Overall, teams entered the simulation with relatively low to moderate familiarity, with no significant difference between groups (control: 3.20 ± 0.691; experimental: 2.77 ± 0.507; *p* = 0.241).

### Performance

The cumulative non-technical performance score for the control groups was 1.8 ± 0.4 and for the experimental group it was 1.6 ± 0.5; *p* = 0.349. Control and experimental groups both received the same score (1.8 ± 0.4; *p* = 0.965) for their technical components.

### Confidence self-reports

Student self-reflections showed similarly high levels of mastering simulation content and acquiring clinically relevant skills (control: 4.0 ± 0.37; experimental 4.0 ± 0.38). Scores were lowest for Question 8 across both groups, with the average score around 2, indicating the students tend to view the instructor as primarily responsible for identifying essential learning content during the simulation.

### Epistemic Network Analysis (ENA)

**Fig. 2:**
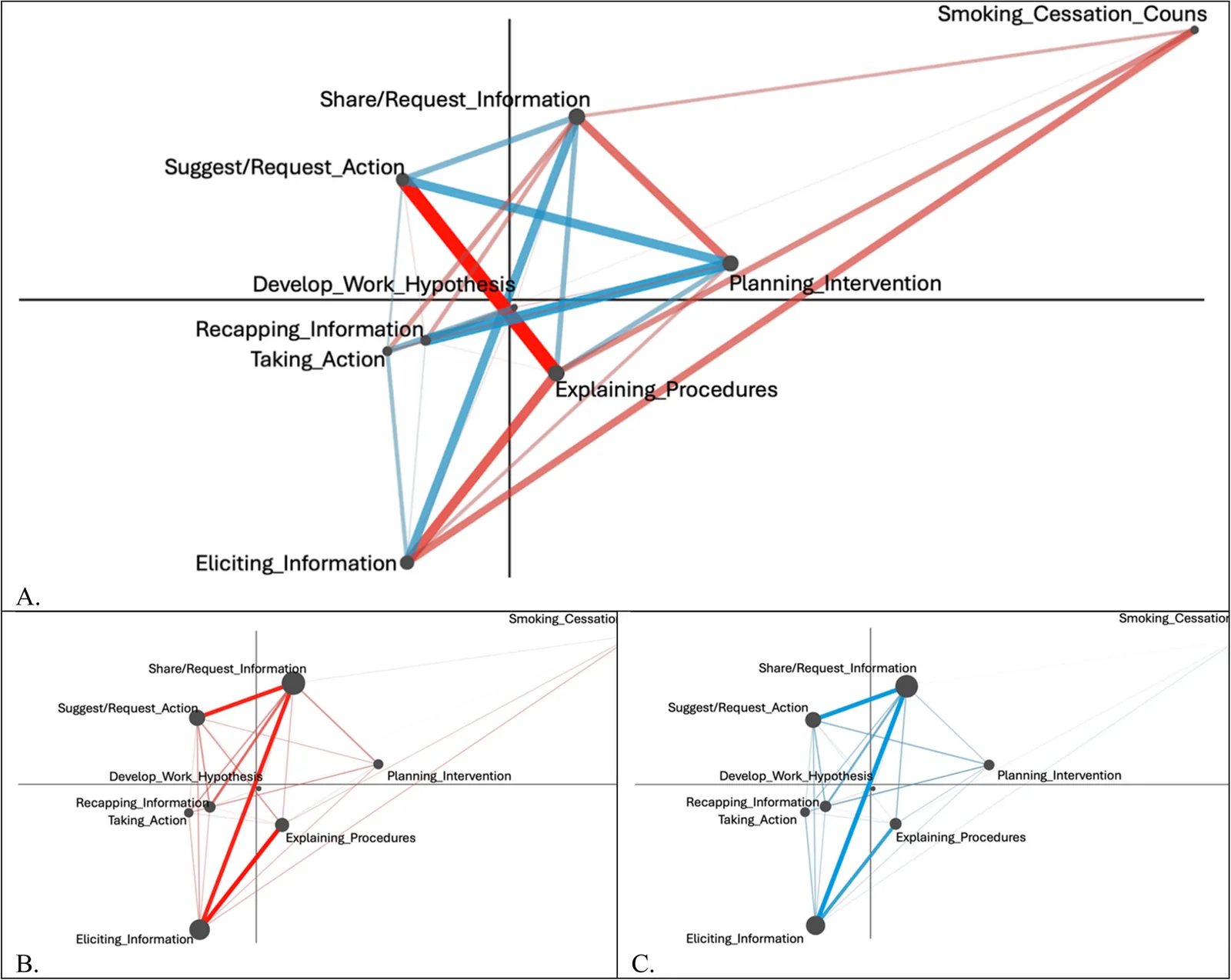
**A** Difference ENA network – control group red, experimental group blue. Black nodes represent the codes, and thicker, more saturated lines between the nodes represent stronger connections. **B** Mean ENA network for the control group. **C** Mean ENA network for the Experimental group

ENA was conducted to examine differences in the structure of teams’ collaborative clinical reasoning during the second simulation. A nonparametric Wilcoxon rank-sum test showed that teams in the experimental condition (Mdn = -0.11, *N* = 6) differed significantly (α = 0.05 level) from teams in the control condition (Mdn = 0.08, *N* = 6, U = 33.00, *p* = 0.02, *r*=-0.83). Across both conditions, the strongest associations in the discourse network occurred between *Explaining Procedures ↔ Eliciting information*,* Eliciting information ↔ Share/Request information*,* Share/Request information ↔ Suggest/Request action*. Few associations were observed between codes related to smoking cessation and planning and intervention, including *Planning Intervention* and *Taking action* (Fig. 2).

ENA subtraction network analysis revealed differences in the associations between conditions (Fig. 2). In the experimental condition, stronger connections were observed between *Suggest/Request action*,* Planning Intervention*, and *Taking action*, indicating more frequent co-occurrences of planning and action-oriented statements during the simulation. In the control condition, the network analysis showed stronger associations between *Explaining Procedures*,* Eliciting Information*, and *Share/Request information*, with comparatively weaker links connecting the planning and action-related codes. We included a sample of coded utterances from the simulation transcripts in Supplementary Material 1: Appendix C. These raw data examples illustrate meaningful differences in the diagnostic and intervention reasoning between the teams in two conditions. Concretely, the network differences suggest that a structured reflective pause coupled with the PARSE framework supports student teams in enacting the “*Strategize Prevention*” and “*Execute Response*” practices by moving more effectively from information gathering toward coordinated clinical management action during the simulated emergency scenario.

## Discussion

This study explored how incorporating a structured reflective pause influenced the technical practices and collaborative clinical reasoning of second-year dental students during a team-based emergency simulated scenario. Outcome measures, including expert-rated technical and non-technical performance scores and post-simulation self-reported confidence scores, did not show differences between the experimental and control groups. However, process measures using epistemic network analysis revealed improvements in how teams in the experimental condition coordinated and applied learned concepts during the subsequent simulation. These findings revealed that structured reflective pauses improve the quality of student teams’ diagnostic and intervention clinical reasoning skills, despite no immediate differences as measured by traditional third-party observational ratings.

### Reflection-through-action enhances translation between diagnostic reasoning and intervention reasoning

The mean ENA networks shared several strong connections across conditions, these similarities were expected given that teams completed the same curricular preparation, participated in the same respiratory and cardiovascular scenarios, and responded to the same patient clinical presentations. The strongest common connections reflected behaviors generally required by the case, including eliciting patient information, explaining procedures, and requesting or sharing information among team members. However, the meaningful interpretation of the ENA results lies in the specific connections that differentiated the networks, rather than broad differences across the entire network structure. Concretely, ENA analysis of teams in the control condition showed a weak coupling between *Planning Intervention* and *Taking action*. The students in these teams tended to focus more on procedural explanations, tangential questions to the patient, or disconnected suggestions that were not integrated into a cohesive diagnostic or management plan. In contrast, teams in the experimental condition showed significantly more frequent associations between *Suggest/Request action*, *Planning Intervention* and *Taking action*, revealing a coupling between proposed diagnostic interventions and their subsequent execution into clinical plans and actions. Experimental teams pursued more purposeful lines of patient history questioning, linking diagnostic reasoning directly to treatment decisions. A care team may ask appropriate questions or suggest possible actions, but those behaviors have limited educational and clinical impact if they remain disconnected from intervention planning and execution. This pattern supports prior work suggesting that reflection-through-action facilitates the formation of ongoing evaluation and planning processes that underlie metacognitive monitoring and intentional decision-making under uncertainty [12, 29].

The PARSE framework applied in the experimental group functioned as intended by prompting teams to anticipate clinical risks and to move from the information gathering and hypothesis sharing towards the clinical planning and action phases. The ENA findings demonstrate that reflection-through-action does not simply increase the *frequency* of desirable behaviors but reshapes the *structure* of collaborative clinical reasoning. Increased coordination between patient history, clinical plans, and actions are indicators of more integrated collaborative clinical reasoning, in which teams consistently translate gathered information into directed decision making. These findings align with prior work by Lee et al., demonstrating that reflective pauses, faculty feedback, and an adaptation period during simulation decrease individual cognitive load and increase patient status awareness, thus increasing learner performance [25]. These patterns align with prior studies on team reflexivity, in which structured transition phases support collective information-processing and strategy adjustment during task execution [23, 43, 44]. Applying the PARSE framework across a wide range of clinical simulations may help amplify the formation of adaptive diagnostic and intervention processes in high-stakes clinical contexts marked by uncertainty.

### ENA as a tool for identifying near and immediate transfer collaborative clinical reasoning practices

Despite observed differences in reasoning processes, expert-rated technical and non-technical performance scores did not significantly differ between groups. This finding is consistent with prior simulation research showing that short, single-session educational interventions may not yield immediate differences in externally scored performance [28, 45], particularly when baseline performance is already high or scoring rubrics lack granularity [46].

However, ENA offers a more granular analysis showing discernible differences in how teams structured and translated differential diagnoses into action-directed decisions. This suggests that third-party observational scoring systems, which tend to emphasize task completion rather than internal or collaborative clinical reasoning processes, consistently do not capture differences observed through discourse analysis [47, 48].

The post-simulation self-reported confidence ratings failed to detect differences across groups. This may have been due to uniformly high scores across groups, which could limit the sensitivity of self-report measures in capturing perceived learning gains.

Prior reflection-based educational studies have largely centered around in-simulation performance [25], with limited attention to the transfer of enhanced clinical decision-making to a follow-up scenario. The present study extends this work by assessing near and proximal transfer across two sequential simulations and allows for the visualization of which reasoning processes were carried forward and applied. Together, these findings underscore the value of analytic approaches, such as ENA, for capturing subtler transferable reasoning processes [49, 50].

### Educational and patient safety implications

From an educational standpoint, these findings suggest that incorporating brief, structured reflective pauses into simulation-based curricula may enhance students’ clinical skills including organizing information, aligning team understanding, and applying diagnostic and intervention reasoning into coordinated action. For dental trainees in particular, emergency scenarios are infrequent but high stakes, making cognitive preparedness and team coordination essential for patient safety.

The PARSE framework and guided reflection questions used in this study provide a practical, low-resource approach that can be integrated into existing simulation curricula without extending total instructional time. PARSE also offers a shared language for teams and instructors to make clinical reasoning visible and therefore coachable. By succinctly summarizing key history or vital signs, anticipating likely deterioration of the patient’s condition, and agreeing on a role assignment before execution, learners can translate their assessment into a coordinated action plan while reducing omissions and improving communication under pressure. By encouraging anticipatory thinking, contingency planning, and shared mental model formation, reflection-through-action may better prepare learners for unpredictable clinical deterioration, where rapid yet deliberate decision-making is required.

Findings from this study have also informed subsequent instructional practice in a number of courses at the University of Michigan School of Dentistry and the PARSE framework has been incorporated beyond the original simulation activity. It is now being used in the respiratory course case-based discussion and as part of a clinical pilot focused on implementing professional activities in predoctoral dental education. These applications are intended to reinforce students’ ability to systematically assess patient risk, anticipate and recognize potential emergencies, develop prevention strategies, and execute appropriate responses in clinical settings. In this way, the study supported curricular refinement by identifying PARSE as a structured approach for making clinical reasoning, teamwork, communication, role clarity, and patient safety more explicit within dental education.

### Limitations

Several limitations must be considered when interpreting these findings. Team level analyses, with reduced statistical power, may have contributed to obscuring performance assessments and attenuated individual-level learning effects. Large team sizes exacerbated this limitation, as verbal contributions were distributed unevenly, with quieter students less likely to articulate their reasoning during simulations. Future studies should employ multilevel modeling, for example, mixed-effects models with random intercepts for teams to measure variance at the individual and team levels. The small number of teams in the present study (*n* = 12) rendered this approach underpowered, but replication studies with larger team samples should consider multilevel analytic approaches (e.g., HINA by Feng et al. [51], transmodal analysis by Shaffer et al. [52], or MOVIE by Canché [53]).

Although overall pre-simulation knowledge scores were comparable across groups, the experimental group had higher baseline cardiovascular-specific knowledge, potentially affecting the magnitude of the network differences observed. Also, the performance rubric relied on observable behaviors and global actions typical of conventional simulation assessment, which did not differ between conditions. However, global measures may inadequately capture learning effects, as they emphasize manifest over latent performance variables [47]. This limitation is consistent with prior simulation research demonstrating that evidence of learning does not always translate into measurable differences in observable performance [45], and aligns with broader concerns that direct observation tools fail to assess non-observable constructs such as clinical reasoning depth, cognitive load, or decision-making processes that underlie team and individual performance [47, 48].

Variability in instructor facilitation and roles may have influenced team behaviors, including scenario responses and simulation duration. Although instructors were provided with a standardized guide, differences in questioning style, information delivery, and situational judgments regarding when the simulation endpoint, specifically contacting emergency medical services, had been reached likely introduced variability across teams. To minimize facilitator-related effects, future implementations of these learning scenarios must have consistent and formalized instructor training, supported by more explicitly defined scripts and objective decision thresholds, thereby reducing reliance on subjective instructor interpretation [54].

Finally, as the study was conducted with dental students at a single institution, generalizability is limited. Future work should examine the application of reflection-through-action frameworks across institutions, professions, and clinical contexts.

## Conclusion and future directions

This study contributes to the clinical simulation literature by highlighting the value of instructor-provided structured reflection-through-action as a mechanism for fostering collaborative diagnostic and intervention clinical reasoning. While traditional learning outcome measures did not reveal significant differences between conditions, ENA results showed that structured reflective pauses were associated with more frequent connections between planning and action during teams’ subsequent emergency simulation performance. This demonstrates a novel effect of reflective pauses, coupled with the PARSE framework, in supporting the translation of diagnostic reasoning into intervention planning and execution, as well as the near and proximal transfer of these reasoning processes in dental students. These findings highlight the importance of examining not only how students perform, but how they think and reason while engaging in clinical scenarios. Future research should examine whether reflection-through-action supports far transfer to dissimilar clinical contexts and distal transfer over longer time intervals, beyond the immediate post-intervention period examined here.

## Supplementary Information

Supplementary Material 1.

## Data Availability

No datasets were generated or analysed during the current study.

## References

[1] Lee JY, Szulewski A, Young JQ, Donkers J, Jarodzka H, van Merrienboer JJG. The medical pause: Importance, processes and training. Med Educ. 2021;55(10):1152–60. 10.1111/medu.14529 PMC8518691 33772840

[2] Reale C, Salwei ME, Militello LG, Weinger MB, Burden A, Sushereba C, et al. Decision-Making During High-Risk Events: A Systematic Literature Review. J Cogn Eng Decis Mak. 2023;17(2):188–212. 10.1177/15553434221147415 PMC10564111 37823061

[3] Rosen MA, DiazGranados D, Dietz AS, Benishek LE, Thompson D, Pronovost PJ, et al. Teamwork in healthcare: Key discoveries enabling safer, high-quality care. Am Psychol. 2018;73(4):433–50. 10.1037/amp0000298 PMC6361117 29792459

[4] Higgins D, Hayes M, Taylor J, Wallace J. A scoping review of simulation-based dental education. MedEdPublish (2016). 2020;9:36. 10.15694/mep.2020.000036.1 PMC10697570 38058871

[5] Sawyer T, Eppich W, Brett-Fleegler M, Grant V, Cheng A. More Than One Way to Debrief: A Critical Review of Healthcare Simulation Debriefing Methods. Simul Healthc. 2016;11(3):209–17. 10.1097/SIH.0000000000000148 27254527

[6] Constanze Richters MS, Anika Radkowitsch F, Behrmann M, Weidenbusch MR, Fischer R, Schmidmaier F, Fischer. Fostering collaboration in simulations: How advanced learners benefit from collaboration scripts and reflection. Learn Instruction. 2024;93. 10.1016/j.learninstruc.2024.101912.

[7] Campbell F, Rogers H. Through the looking glass: a review of the literature surrounding reflective practice in dentistry. Br Dent J. 2022;232(10):729–34. 10.1038/s41415-022-3993-4 PMC9137255 35624264

[8] McMullen M, Wilson R, Fleming M, Mark D, Sydor D, Wang L, et al. Debriefing-on-Demand: A Pilot Assessment of Using a Pause Button in Medical Simulation. Simul Healthc. 2016;11(3):157–63. 10.1097/SIH.0000000000000140 26953566

[9] Keiser NL, Arthur W. A meta-analysis of the effectiveness of the after-action review (or debrief) and factors that influence its effectiveness. J Appl Psychol. 2021;106(7):1007–32. 10.1037/apl0000821 32852990

[10] Mamede S, van Gog T, Sampaio AM, de Faria RM, Maria JP, Schmidt HG. How can students’ diagnostic competence benefit most from practice with clinical cases? The effects of structured reflection on future diagnosis of the same and novel diseases. Acad Med. 2014;89(1):121–7. 10.1097/ACM.0000000000000076 24280855

[11] Mulli J, Nowell L, Lind C. Reflection-in-action during high-fidelity simulation: A concept analysis. Nurse Educ Today. 2021;97:104709. 10.1016/j.nedt.2020.104709 33352351

[12] Schmutz JB, Lei Z, Eppich WJ. Reflection on the Fly: Development of the Team Reflection Behavioral Observation (TuRBO) System for Acute Care Teams. Acad Med. 2021;96(9):1337–45. 10.1097/ACM.0000000000004105 33788785

[13] Schon DA. The Reflective Practitioner: How Professionals Think In Action. New York: Basic Books; 1983. p. 384.

[14] Schön DA. Educating the reflective practitioner: Toward a new design for teachingand learning in the professions. San Francisco: Jossey-Bass; 1987.

[15] Popov V, Harmer B, Raphael S, Scott I, Sample AP, Cooke JM, Cole M. Elucidating cognitive processes in cardiac arrest team leaders: a virtual reality-based cued-recall study of experts and novices. Annals of medicine. 2025;57(1):2470976. 10.1080/07853890.2025.2470976. PMC11878170 40028867

[16] DeShon RP, Kozlowski SW, Schmidt AM, Milner KR, Wiechmann D. A multiple-goal, multilevel model of feedback effects on the regulation of individual and team performance. J Appl Psychol. 2004;89(6):1035–56. 10.1037/0021-9010.89.6.1035 15584840

[17] Vitaliy Popov CG. Sarah Tomaka & Theodora Danciu. Making the invisible visible: how multisource feedback and guided facilitation affect team reflection. Cogn Technol Work. 2026;28:135–54. 10.1007/s10111-025-00835-4.

[18] Michaéla C, Schippers MAW, Jeremy F. Dawson. Team reflexivity and innovation: the moderating role of team context. South Manage Association. 2012;41(3).

[19] Schmutz JB, Eppich WJ. Promoting Learning and Patient Care Through Shared Reflection: A Conceptual Framework for Team Reflexivity in Health Care. Acad Med. 2017;92(11):1555–63. 10.1097/ACM.0000000000001688. 28445215

[20] West MA. Reflexivity, Revolution, and Innovation in Work Teams. In: Michael M, Beyerlein DAJ, Beyerlein ST, editors. Product Development Teams. Volume 5. Stanford, USA: JAI; 2000. pp. 1–29.

[21] Tschan F, Keller S, Semmer NK, Timm-Holzer E, Zimmermann J, Huber SA, et al. Effects of structured intraoperative briefings on patient outcomes: multicentre before-and-after study. Br J Surg. 2021;109(1):136–44. 10.1093/bjs/znab384 PMC10401893 34850862

[22] Keller S, Tschan F, Semmer NK, Trelle S, Manser T, Beldi G. StOP? II trial: cluster randomized clinical trial to test the implementation of a toolbox for structured communication in the operating room-study protocol. Trials. 2022;23(1):878. 10.1186/s13063-022-06775-y PMC9580155 36258223

[23] Marks MA, John E, Mathieu, Stephen J. Zaccaro. A Temporally Based Framework and Taxonomy of Team Processes. Acad Manage Rev. 2001;26(3):356–76.

[24] Fanning RM, Gaba DM. The role of debriefing in simulation-based learning. Simul Healthc. 2007;2(2):115–25. 10.1097/SIH.0b013e3180315539 19088616

[25] Lee JY, Donkers J, Jarodzka H, Sellenraad G, Faber TJE, van Merrienboer JJG. The Effects of Reflective Pauses on Performance in Simulation Training. Simul Healthc. 2024;19(2):82–9. 10.1097/SIH.0000000000000729 37094368

[26] Sullivan S, Ruis A, Pugh C. Procedural Simulations and Reflective Practice: Meeting the Need. J Laparoendosc Adv Surg Tech A. 2017;27(5):455–8. 10.1089/lap.2016.0639 28157421

[27] Clapper TC, Leighton K. Incorporating the Reflective Pause in Simulation: A Practical Guide. J Contin Educ Nurs. 2020;51(1):32–8. 10.3928/00220124-20191217-07 31895468

[28] Heyn LG, Brembo EA, Byermoen KR, Cruaud C, Eide H, Flo J, et al. Exploring facilitation in virtual simulation in nursing education: A scoping review. PEC Innov. 2023;3:100233. 10.1016/j.pecinn.2023.100233 PMC10687044 38033419

[29] Mamede S, Schmidt HG. Deliberate reflection and clinical reasoning: Founding ideas and empirical findings. Med Educ. 2023;57(1):76–85. 10.1111/medu.14863 PMC10083910 35771936

[30] Anderson JM, Aylor ME, Leonard DT. Instructional design dogma: creating planned learning experiences in simulation. J Crit Care. 2008;23(4):595–602. 10.1016/j.jcrc.2008.03.003 19056028

[31] Zhong LA, Chen KY, Hsieh MJ, Ho MC. Near and far transfer effects of working memory training: A preregistered, double-blind, randomized-controlled study. Acta Psychol (Amst). 2025;260:105499. 10.1016/j.actpsy.2025.105499 40914122

[32] Gachter S, Starmer C, Tufano F. Measuring the Closeness of Relationships: A Comprehensive Evaluation of the ‘Inclusion of the Other in the Self’ Scale. PLoS ONE. 2015;10(6):e0129478. 10.1371/journal.pone.0129478. PMC4466912 26068873

[33] Yule S, Flin R, Paterson-Brown S, Maran N. Non-technical skills for surgeons in the operating room: a review of the literature. Surgery. 2006;139(2):140–9. 10.1016/j.surg.2005.06.017 16455321

[34] Jeffries PR, Rizzolo MA. Designing and Implementing Models for the Innovative Use of Simulation to Teach Nursing Care of Ill Adults and Children: A National, Multi-Site, Multi-Method Study. National League of Nursing; 2006.

[35] Jeffries PR. Simulation in Nursing Education: From Conceptualization to Evaluation. National League for Nursing; 2012. p. 288.

[36] Franklin AE, Burns P, Lee CS. Psychometric testing on the NLN Student Satisfaction and Self-Confidence in Learning, Simulation Design Scale, and Educational Practices Questionnaire using a sample of pre-licensure novice nurses. Nurse Educ Today. 2014;34(10):1298–304. 10.1016/j.nedt.2014.06.011 25066650

[37] Cress U. The Need for Considering Multilevel Analysis in CSCL Research—An Appeal for the Use of More Advanced Statistical Methods. Int J Computer-Supported Collaborative Learn. 2008;3(1):69–84.

[38] DW S. Epistemic network analysis: Understanding learning by using big data for thick description. In: Fischer FH-SC, Goldman SR, Reimann P, editors. International handbook of the learning sciences. New York: Routledge; 2018.

[39] Popov V, Tan Y, Manojlovich M. Applying ordered network analysis to video-recordedphysician–nurse interactions to examine communication patterns associated with shared understanding in inpatient oncology care settings. BMJ open. 2024;14(6):e084653. 10.1136/bmjopen-2024-084653 PMC11191829 38889940

[40] Ruis A. Finding common ground. Proceedings of the 13th International Conference on Computer Supported Collaborative Learning (CSCL 2019)2019.

[41] Smagorinsky P. The method section as conceptual epicenter in constructing social science research reports. Writ Communication. 2008;25(3):389–411. 10.1177/0741088308317815.

[42] Psycholinguistics, MPIf. ELAN. 7.0 ed. Nijmegen, Netherlands: The Language Archive; 2025.

[43] Schmutz JB, Lei Z, Eppich WJ, Manser T. Reflection in the heat of the moment: The role of in-action team reflexivity in health care emergency teams. J Organizational Behav. 2018;39(6):749–65.

[44] Schippers MC, Edmondson AC, West MA. Team reflexivity. In: Argote LL, John M, editors. The Oxford Handbook of Group and Organizational Learning. New York, NY: Oxford University Press; 2020. pp. 175–94.

[45] Ilgen JS, Sherbino J, Cook DA. Technology-enhanced simulation in emergency medicine: a systematic review and meta-analysis. Acad Emerg Med. 2013;20(2):117–27. 10.1111/acem.12076 23406070

[46] Colman N, Wiltrakis SM, Holmes S, Hwu R, Iyer S, Goodwin N, et al. A comparison of rapid cycle deliberate practice and traditional reflective debriefing on interprofessional team performance. BMC Med Educ. 2024;24(1):122. 10.1186/s12909-024-05101-1 PMC10848365 38326900

[47] Sebok-Syer SS, Tawfik D. We cannot measure what we have not yet attempted to measure: Steps towards reconceptualising team-based assessments in the health professions. Med Educ. 2025;59(9):895–7. 10.1111/medu.15762 40619930

[48] Kolbe M, Boos M. Laborious but Elaborate: The Benefits of Really Studying Team Dynamics. Front Psychol. 2019;10:1478. 10.3389/fpsyg.2019.01478 PMC6611000 31316435

[49] Popov V, Rochlen LR. From communication to action: using orderednetwork analysis to model team performance in clinical simulation. BMC medical education. 2025;25(1):479. 10.1186/s12909-025-07062-5 PMC11967124 40175985

[50] Rochlen LR, Putnam EM, Tait AR, Du H, Popov V. Sequential behavioral analysis: a novel approach to help understand clinical decision-making patterns in extended reality simulated scenarios. Simulation in Healthcare. 2023;18(5):321-5. 10.1097/SIH.0000000000000686 PMC9989035 36111990

[51] Feng SH, Gasevic B, Kirkley D. A. Heterogeneous Interaction Network Analysis (HINA): A new learning analytics approach for modelling, analyzing, and visualizing complex interactions in learning processes2026; arXiv:2601.06771.

[52] Shaffer DWW, Ruis Y. Transmodal analysis. J Learn Analytics. 2025;12(1):271–92. 10.18608/jla.2025.8423.

[53] González Canché MS. Mapping, organizing, and visualizing interdependent events (MOVIE): A rigorous analytic framework and cost-free software application designed to model temporal and dynamic complex realist structures in social research settings. Methodological Innovations. 2022;15(3):263–88.

[54] Dieckmann P, Molin Friis S, Lippert A, Ostergaard D. The art and science of debriefing in simulation: Ideal and practice. Med Teach. 2009;31(7):e287–94. 10.1080/01421590902866218 19811136

