# Supplementary material for: Effects of reflection-through-action on collaborative clinical reasoning skills in an emergency dental simulation
Source: Adv Simul (Lond). 2026 Jul 27;11:78. doi: 10.1186/s41077-026-00463-6 (PMC13693120; doi:10.1186/s41077-026-00463-6)
Supplement: Supplementary file 1 [file 41077_2026_463_MOESM1_ESM.docx]

**Appendix A**

**PARSE**

**P - Patient history/vitals**

**A - Anticipate risks**

**R - Recognize symptoms**

**S - Strategize prevention**

**E - Execute response**

| **Pre-appointment** | **Patient: history and vital signs** | **Anticipate it!** | **Recognize it!** | **Strategize prevention** | **Execute response** |
| --- | --- | --- | --- | --- | --- |
| Review patient’s:   - Medical history - Social history - Medication - Chart to confirm procedure | - Update medical history and medications - Determine if there has been a deterioration in medical status - Based on info from history, vital signs, other signs and symptoms, dental procedure, decide: is this patient safe to treat?   Ex: patient has Type I Diabetes Mellitus and he has daily steroid injections.  The patient is safe to treat b/c his diabetes is well-controlled (last HbA1c:4), he took insulin, and ate. | - What would make this patient have a medical emergency?   Ex: Hypoglycemia—the patient takes his insulin but skips breakfast; dental appt is at 8 am | - What are the signs and symptoms that can alert you that the patient is approaching a medical emergency?   Ex: Looking pale, sweating, tremor, irritability, etc | - How can you prevent this medical emergency?   Ex: Check that patient took his insulin and ate breakfast (dental appt is at 8 am) | - How can I address this potential emergency?   Ex: give patient glucose (orange juice or other)—this means that you need to be prepared for this emergency situation by having the glucose supplement nearby. |
| **TEAMWORK-FOCUSED:** What should we do differently or focus on next time in terms of teamwork (e.g., communication, seeking/offering help, role clarity, information sharing)? Be clear about who will do what and how this will help our team be successful in the next simulation case? | | | | | |

**Appendix B**

**Pre- and Post-Test Questions**

Quiz: pre- and post-simulation

| **Question** | **Answer and justification** | **Place in simulation** |
| --- | --- | --- |
| Which of the following spirometry findings after the administration of a bronchodilator is consistent with a diagnosis of COPD?  A) FEV1/FVC ratio > 0.8  B) FEV1 < 80% predicted  C) FVC < 80% predicted  D) FEV1/FVC ratio < 0.7 | D  Diagnosis of COPD is based on spirometry findings showing a post-bronchodilator FEV1/FVC ratio < 0.7. | Respiratory  2B—students receive the patient’s pulmonary function tests and need to interpret them |
| All of the following except one have been implicated on the development of COPD. Which is the EXCEPTION?  A) Smoking  B) Occupational exposure to dust and chemicals  C) Family history of asthma  D) Alpha-1 antitrypsin deficiency | C  While smoking, occupational exposure to dust and chemicals, and alpha-1 antitrypsin deficiency are all well-established risk factors for COPD, family history of asthma is not considered a risk factor. | Respiratory  Based on the information students received pre-simulation, they should link the patient’s significant smoking history with COPD and they should elicit this diagnosis from the patient’s history. |
| A 70-year-old patient with a history of COPD presents to the emergency department with increased dyspnea, cough, and purulent sputum production. On examination, the patient is febrile with increased respiratory rate and bilateral coarse crackles on auscultation. Which of the following is the most likely explanation for the patient's worsening symptoms?  A) Development of albuterol resistance  B) Acute exacerbation of COPD  C) Development of pneumonia  D) Pulmonary embolism | C  Patients with COPD are at increased risk of developing respiratory tract infections, such as pneumonia, which can lead to worsening of symptoms including increased dyspnea, cough, and sputum production. | Respiratory  2—ddx discussion that includes a respiratory infection |
| Which of the following characteristics helps distinguish angina from myocardial infarction (MI)?  A) Pain radiating to left jaw  B) Pain worsened by deep breathing  C) Pain associated with diaphoresis and nausea  D) Pain aggravated by chest wall palpation | C  While both angina and myocardial infarction (MI) can present with chest pain, the presence of symptoms such as diaphoresis and nausea is more indicative of an MI | Cardiovascular |
| What is the primary mechanism of action of nitroglycerin in the management of angina?  A) Inhibition of platelet aggregation  B) Vasodilation through stimulation of beta receptors  C) Activation of guanylate cyclase leading to increased cyclic GMP  D) Inhibition of angiotensin-converting enzyme (ACE) | C  Nitroglycerin is a nitric oxide donor that activates guanylate cyclase, leading to increased cyclic GMP (cGMP) levels, which in turn causes vasodilation of coronary arteries, resulting in improved myocardial perfusion and relief of angina symptoms. | Cardiovascular |

**Appendix C**

**Sample Coded Utterances from Simulation Transcripts**

***All student names changed to protect anonymity**

Control (Team 3): example highlighting Eliciting Information and Suggest/Request Information

| **Student*** | **Utterance** | **Sub-Code** |
| --- | --- | --- |
| Gary | Are we just using the nitroglycerin? | **Request/Share Information** |
| Irina | Can we have him chew a baby aspirin, like I don't know? | **Suggest/Request Action** |
| Darcy | Ok you know what, actually- actually the- the thing ran out, we're gonna give him some- some tablets under his tongue. | **Taking Action** |
| Irina | Nitroglycerin? | **Request/Share Information** |
| Darcy | Of, yeah fast release nitroglycerin. | **Request/Share Information** |
| Irina | Did you hear that, Mr. Angus? | **Eliciting Information** |
| Darcy | How's that? | **Eliciting Information** |
| Darcy | Do you feel that- does that feel any better? | **Eliciting Information** |
| Gary | Ooh his heart rate- his heart rate lowered. | **Request/Share Information** |
| Felix | Goin down? | **Request/Share Information** |
| Gary | Isn't it? | **N/A** |
| Darcy | To twelve. | **Request/Share Information** |
| Gary | Wasn't it 110, or is that just... | **Request/Share Information** |
| Irina | (unintelligible). | **N/A** |
| Felix | Let's recheck the blood pressure. | **Suggest/Request Action** |
| Darcy | Hi Mr. Angus do you still feel like an elephant's on top of you? | **Eliciting Information** |
| Henry | Here, take the blood pressure. | **Taking Action** |
| Irina | (unintelligible), I'm not left handed, I can't really do this. | **Request/Share Information** |
| Gary | Try your right hand. | **Suggest/Request Action** |
| Irina | It's gonna be like... | **N/A** |
| Benjamin | Does your heart chest still hurt? | **Eliciting Information** |
| Irina | Did you have any improvement in symptoms after administration of the nitroglycerin? | **Eliciting Information** |

Control (Team 6): example highlighting relationship between Eliciting Information, Request/Share Information and Explaining Procedures

| **Student*** | **Utterance** | **Sub-Code** |
| --- | --- | --- |
| George | Does a specific side of your chest hurt or is it all over? | **Eliciting Information** |
| Ian | Okay, 182, put it under the- okay go ahead and put this under your tongue. | **Explaining Procedures** |
| George | Oh no 180, it went up, it went up. | **Request/Share Information** |
| Ian | This is nitroglycerin. | **Explaining Procedures** |
| Donald | Relieve your chest pain. | **Explaining Procedures** |
| Ian | I thought you take it at home. | **N/A** |
| Bailey | The nitroglycerin will open up your [UM] arteries and- or vasodilate them, and you should be able to breathe better. | **Recapping Information** |
| Ian | No we- we used limited amount of [UH] epinepherine, so it shouldn't be from the anesthesia. | **Recapping Information** |
| Ian | Okay, go ahead. | **Planning Intervention** |
| Ian | Alright, we waited for five minutes after you've taken the medication, we're gonna take your blood pressure again. | **Explaining Procedures** |
| Emily | [UM]. | **N/A** |
| Ian | Alright, how you feeling to- right now Mr. Angus [patient’s name], much better? | **Eliciting Information** |
| Donald | What's not feeling good? | **Eliciting Information** |
| Cameron | What hurts? | **Eliciting Information** |
| ian | Alright, I'm gonna check your- your pulse. | **Explaining Procedures** |
| Emily | Is it your whole chest that hurts, or is it a specific part of your chest? | **Eliciting Information** |
| Cameron | In his chest. | **N/A** |
| Emily | Just all over, (unintelligible). | **N/A** |
| Bailey | Maybe he aspirated. | **Develop Working Hypothesis** |
| Cameron | You hear anything [student name]? | **Request/Share Information** |
| Ian | Not really (unintelligible). | **Request/Share Information** |
| Emily | Breathing sounds fine? | **Request/Share Information** |
| George | Are we unresponsive? | **Request/Share Information** |
| Cameron | Maybe if their resp- their respiratory rate's fine. | **Request/Share Information** |
| Florian | Mr. Angus is talking through the thing...I- I think he's talking still. | **Request/Share Information** |
| Cameron | Heart rate is good, we're losing oxygen. | **Request/Share Information** |

Experimental (Team 10): example highlighting Suggest/Request Action, Planning Intervention and Taking Action

| **Student*** | **Utterance** | **Sub-Code** |
| --- | --- | --- |
| Annie | Your chest hurts? | **Eliciting Information** |
| Frank | We'll get you some nitroglycerin | **Taking Action** |
| Annie | And we'll sit you up | **Taking Action** |
| Frank | Are you feeling a little bit cold? | **Eliciting Information** |
| Frank | A little light-headed? | **Eliciting Information** |
| Geoff | Well [UM], we should measure his glucose | **Planning Intervention** |
| Geoff | We're gonna measure your glucose real quick and then we're gonna get someone to grab [UM] some glucose tabs or glucose gel | **Taking Action** |
| Annie | Yeah, I don't think that would cause chest pain | **Recapping Information** |
| Frank | Here's your nitroglycerin | **Taking Action** |
| Frank | Are you starting to feel better now? | **Eliciting Information** |
| Frank | Okay, let's call for the ambulance | **Planning Intervention** |
| Danielle | I'm gonna call. | **Taking Action** |
| Geoff | We're gonna go call an ambulance | **Taking Action** |
| Geoff | It's okay | **N/A** |
| Frank | Do you need some oxygen in the meantime? | **Request/Share Information** |
| Danielle | Yes we do give some oxygen | **Request/Share Information** |
| Annie | Was the tooth, do you feel anything stuck in the back of your throat? | **Eliciting Information** |
| Annie | You just feel pain like you would when you have angina? | **Eliciting Information** |
| Frank | The ambulance is on the way | **Request/Share Information** |

Experimental (Team 11): example highlighting Suggest/Request Action, Planning Intervention and Taking Action

| **Student*** | **Utterance** | **Sub-Code** |
| --- | --- | --- |
| Ben | Reach into his pocket, like get his nitroglycerin out | **Taking Action** |
| Fiona | Yeah | **Taking Action** |
| David | Alright, can we get the vital signs | **Planning Intervention** |
| Ben | That's not good | **Request/Share Information** |
| Ben | Well first off we'd like stop, obviously | **Taking Action** |
| Fiona | Yeah | **Taking Action** |
| Adam | Ask if he's having any pain in his, what, left arm? | **Suggest/Request Action** |
| Fiona | Left arm right? That's heart attack? | **Develop Working Hypothesis** |
| David | Did we tell him to take his nitroglycerin? | **Request/Shae Information** |
| Eugene | Do you have pain anywhere else in your body besides your chest? | **Eliciting Information** |
| Gina | Left jaw, alright | **N/A** |
| Claire | (unintelligible). | **N/A** |
| Claire | Are you able to take your nitroglycerin in the glass bottle right now? | **Taking Action** |
| Ben | (unintelligible). | **N/A** |
| Eugene | So the connection with [UM] | **N/A** |
| David | We're gonna call 911 as well, yeah [UM] just preemptively your nitroglycerin should help this problem | **Explaining Procedures** |
| Fiona | Do we need something else? | **Request/Share Information** |
| Eugene | (unintelligible). | **N/A** |
| Eugene | And what was that pre-quiz thing, that was like the difference between MI and? Yeah but like what was the difference, this is how you tell the difference between myocardial infarction and... angina? | **Develop Working Hypothesis** |
| Fiona | Angina | **N/A** |
| Gina | Angina | **N/A** |
| Eugene | So this would be angina? | **Develop Working Hypothesis** |
| David | I think it's angina | **Develop Working Hypothesis** |
| Gina | Are we calling 911? but the nitroglycerin should have helped | **Suggest/Request Action** |
| David | Calling 911 | **Taking Action** |
